# Advanced ocular surface squamous cell carcinoma (OSSC): long-term follow-up

**DOI:** 10.1007/s00417-021-05264-3

**Published:** 2021-07-20

**Authors:** Gustavo Savino, Giovanni Cuffaro, Martina Maceroni, Monica Maria Pagliara, Maria Grazia Sammarco, Luca Giraldi, Maria Antonietta Blasi

**Affiliations:** 1 Università Cattolica del Sacro Cuore, Rome, Italy; 2 UOC Oncologia Oculare, Fondazione Policlinico Universitario A. Gemelli - IRCCS, Largo Agostino Gemelli, 8, 00168 Rome, Italy; 3 Sezione Di Igiene, Dipartimento Di Scienze Biologiche E Sanità Pubblica, Università Cattolica del Sacro Cuore, Rome, Italy

**Keywords:** Ocular surface squamous carcinoma, Advanced ocular surface squamous neoplasia, Orbit, Exenteration

## Abstract

**Purpose:**

To analyze the clinical characteristics and long-term follow-up of patients with advanced ocular surface squamous cell carcinoma (OSSC) involving periocular tissues and/or orbit. Primary outcomes were overall survival (OS), disease-free survival (DFS), and overall recurrence rate (RR). Secondary outcomes were a correlation between primary outcomes and tumor location, American Joint Committee on Cancer Classification (AJCC) staging system, histological results, surgical margins, and type of treatment. Study design: a retrospective case series.

**Methods:**

The medical records of patients affected by OSSC involving periocular tissues and/or orbit referring, from 01/2011 to 01/2020, to our tertiary referral center were reviewed.

**Results:**

Thirty-six eyes of 36 patients were included. The mean age was 68.2 years; 18 (50%) patients were males. The mean follow-up was 40 months. The RR was 64%. The OS at 12, 24, 36, and 60 months was respectively 97.1%, 92.7%, 92.7%, and 92.7%. The DFS at 12, 24, 36, and 60 months was respectively 62.9%, 50.8%, 41.6%, and 29.7%. Multicentric disease (p = 0.0039), inferior tarsus localization (p = 0.0428), histological diagnosis of high-risk SSCs (p = 0.0264), positive surgical margins (p = 0.0434), and excisional biopsy (EB) alone (p = 0.0005) were associated with an increased risk of recurrence. A shorter OS was observed in patients who underwent EB alone (p = 0.0049).

**Conclusion:**

OSCC involving periocular tissues and/or orbit is an aggressive disease with a high recurrence rate. Multicentric disease, positive surgical margins, inferior tarsus localization, and surgery without adjuvant therapies are strong predictors of recurrence and are the main factors affecting prognosis. 
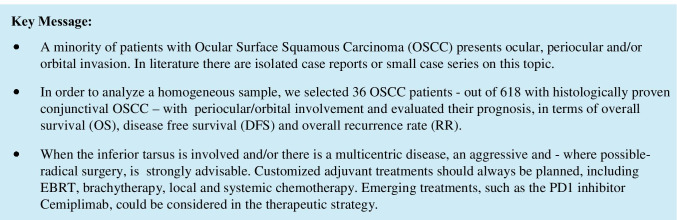

## Introduction

Ocular surface squamous neoplasia (OSSN) includes a spectrum of malignancy that ranges from mild epithelial dysplastic changes (CIN) to more severe invasive carcinoma, invading through the basement membrane into the substantia propria (Ocular surface Squamous Cell Carcinoma - OSCC) [1]. The incidence of OSCC has been reported from 0.03–1.9 per 100,000 persons/year in patients in the USA and Australia, with a higher incidence in males and Whites [2, 3].

Orbital exenteration is probably the treatment of choice for tumors that have invaded the orbit or that exhibit complete involvement of the conjunctiva [4]. Orbital exenteration therefore remains the ultimate therapeutic approach. Nevertheless, very few reports exist on demography, survival disease free, overall survival, and predictive risk factors for OSCC invading periocular tissues and orbit [5].

The scope of this retrospective study was to analyze the prevalence of diffuse OSCC with periocular involvement, the clinical outcomes in relation to the tumors staging, and different medical and surgical approaches. Primary outcomes were overall survival (OS), disease-free survival (DFS) and overall recurrence rate (RR). Secondary outcomes were a correlation between primary outcomes and tumor location, eight editions of the American Joint Committee on Cancer Classification (AJCC) staging system stage, histological results, margins results, and type of treatment.

## Methods

### Patients

Patients suffering from OSCC demonstrating involvement of conjunctiva and adjacent periocular tissues (e.g., eyelid palpebral margin, superior tarsus, inferior tarsus, medial canthus, lateral canthus, orbital tissues) were identified and included in a retrospective study. All patients were treated at the Ocular Oncology Unit of the Fondazione Policlinico Universitario A. Gemelli, Rome, between January 01, 2011, and January 2020. Patients initially received either surgical or topical treatment for their ocular surface disease, followed by subsequent therapies for persistent or recurrent disease. Patients previously medically or surgically treated or with a follow-up period of less than 6 months and or those with missing or incomplete records were excluded. A written informed consent for data collection and analysis of collected data from their medical records was obtained from all patients. The study was carried out with approval from the Head and Neck Institutional Review Board (approval ID; 18/2020) and in adherence to the tenets of the Declaration of Helsinki. Photographs were obtained in selected cases with patients’ permission.

### Outcomes

Overall survival (OS) and overall recurrence rate (RR) were determined along with disease-free survival (DFS). Overall survival was calculated as the time interval between the diagnosis and the last follow-up or death for all causes, if not differently specified. Overall recurrence rate (RR) was defined considering the rate at which the disease recurred in the same or similar site of the original tumor after a complete resolution of the original tumor. DFS was defined as the time from the first treatment to relapse (local recurrence, lymph node metastasis, or distant metastasis) or all-cause death, whichever came first. In addition, the following data were collected: gender, age, site, laterality, size or extent of the primary tumor (T), the presence or absence of lymph node metastases (N) or distant metastases (M), histologic type (carcinoma in situ (CIS) or low-risk carcinoma and invasive squamous cell carcinoma or high-risk SCC), resection boundaries, adjuvant therapies (radiotherapy, chemotherapy, immunotherapy), and duration of follow-up. Tumor localization was defined considering the prevalent involved site; when a prevalent site was not identifiable, the tumor was defined diffuse. The anatomic involvement has been defined as monocentric or multicentric according to whether only one or more anatomic sites were involved: superior tarsus, inferior tarsus, medial canthus, lateral canthus, or orbital tissues.

### Statistics

The Kaplan–Meier method was used to calculate survival rates and to plot survival curves. Analyses were conducted using Stata software (StataCorp. 2015. Stata Statistical Software: Release 15. College Station, TX, USA: StataCorp LP). Relations between categorical variables were analyzed using the log-rank test. Values of p < 0.05 were considered statistically significant.

## Results

During the 9-year study period, there were a total of 36 patients (5.8%), out of 618 total OSSN cases, with excised histologically confirmed SCC involving ocular and periocular tissues recorded. The mean follow-up was 40 months (median: 31 months, range: 6–120 months). Two deaths occurred during the follow-up period, unrelated to periocular malignancy. The mean age at the time of first surgery was 68.2 years (median: 73, range: 33–92). Eighteen (50%) patients were male and 18 (50%) were female. All patients were Caucasians. SCC occurred on the right side in 17 (47%) cases and on the left in 19 (53%). Demographic data are listed in Table 1.

**Table 1:** Demographic, clinical, histological, and treatment features of OSSN patients

Demographic features	
Males (%)/females (%)	18 (50%)/18 (50%)
Age, mean (range)	68.2 (33–92)
Involved eye, RE (%)/LE (%)	17 (47%)/19 (53%)
Involved sites	
Multicentric	21 (58%)
Monocentric	15 (42%)
Inferior eyelid TP	10 (28%)
Superior eyelid TP	6 (17%)
Eyelid margin	6 (17%)
Orbit	5 (14%)
Internal cantus	4 (11%)
External cantus	2 (5%)
Diffuse	3 (8%)
Histology	
Low-risk SCC	5 (14%)
High-risk SCC	24 (67%)
Basaloid SCC	7 (19%)
Positive margins	7 (19%)
Negative margins	29 (81%)
AJCC stage	
T3	31 (86%)
T4a	4 (11%)
T4b	1 (3%)
Treatment	
EB	20 (56%)
EB + (MMC, INF-α)	10 (28%)
IB + (EBRT, MMC, INF-α)	3 (9%)
Exenteratio orbitae	3 (9%)

*AJCC*, American Joint Committee on Cancer; *EB*, excisional biopsy; *EBRT*, external beam radiotherapy; *IB*, incisional biopsy; *INF*, interferon; *LE*, left eye; *MMC*, mitomycin C; *RE*, right eye; *SCC*, squamous cell carcinoma; *TP*, tarsal plate

### Risk categories

Twenty-one patients (58%) showed a multicentric disease, while 15 (42%) cases presented a monocentric disease. The mainly involved site was the inferior eyelid tarsal plate in 10 cases (28%), the superior eyelid tarsal plate in 6 (17%), the eyelid palpebral margin in 6 (17%), orbit in 5 (14%), internal canthus in 4 (11%), and external canthus in 2 (5%). In 3 patients (8%), no prevalent site was identifiable since they were defined as a diffuse disease. Twenty (56%) patients were treated with excisional biopsy alone (EB), 10 (28%) underwent EB plus adjuvant topical therapy (5 with subconjunctival interferon-alpha (INF-α), 4 with mitomycin C (MMC), 1 with both drugs); 3 (8%) cases received external beam radiotherapy (EBRT), MMC, and INF-α respectively after an incisional biopsy; 3 (8%) patients were treated with exenteratio orbitae, one of which with an adjuvant EBRT.

Following histological examination, 5 (14%) cases were classified as low-risk SCCs (carcinoma in situ (CIS)), whereas 24 (67%) were classified as high-risk SCCs (invasive squamous carcinoma); 7 (19%) excised tissues were histologically classified as basaloid SCCs (BSCC). Seven (19%) specimens presented positive margins at the histological examination, while 29 (81%) presented negative surgical margins. The incompletely excised tumors were high-risk SCCs variants in 5 (71%) cases, whereas 2 (29%) were BSCC.

According to the eighth edition of the AJCC, the most common pT category was T3 with 31 (86%) cases, followed by T4a in 4 (11%) and T4b in 1 (3%). Table 1 lists clinical and histological features of OSSN along with the types of treatment.

Overall recurrence rate was 64% (n = 23). Among the 23 recurred patients, 16 presented a multicentric disease (p = 0.0039). The inferior tarsus was associated with the highest recurrence rate (n = 8, p = 0.0428) and high-risk SSCs recurred more frequently than other histologic types (n = 17, p = 0.0264). Positive surgical margins were associated with an increased risk of recurrence n = 5 (p = 0.0434). The highest recurrence rate was observed in the EB group (n = 9, p = 0.0005). Table 2 shows clinical and histological factors significantly associated with recurrence. Five (22%) out of 23 recurred patients presented orbital invasion, manifesting disease progression from the initial T3 stage.

**Table 2:** Clinical and histological factors significantly associated with recurrence

Clinical and histological factors associated with recurrence	P value
Multicentric disease	0.0039
Inferior tarsus	0.0428
High-risk SCC	0.0264
Positive margins	0.0434
EB alone	0.0005

*EB*, excisional biopsy; *SCC*, squamous cell carcinoma

The overall survival rates at 12, 24, 36, and 60 months were respectively 97.1%, 92.7%, 92.7%, and 92.7%.

A short overall survival was observed in patients who underwent EB alone (p = 0.0049).

**Fig. 1:**
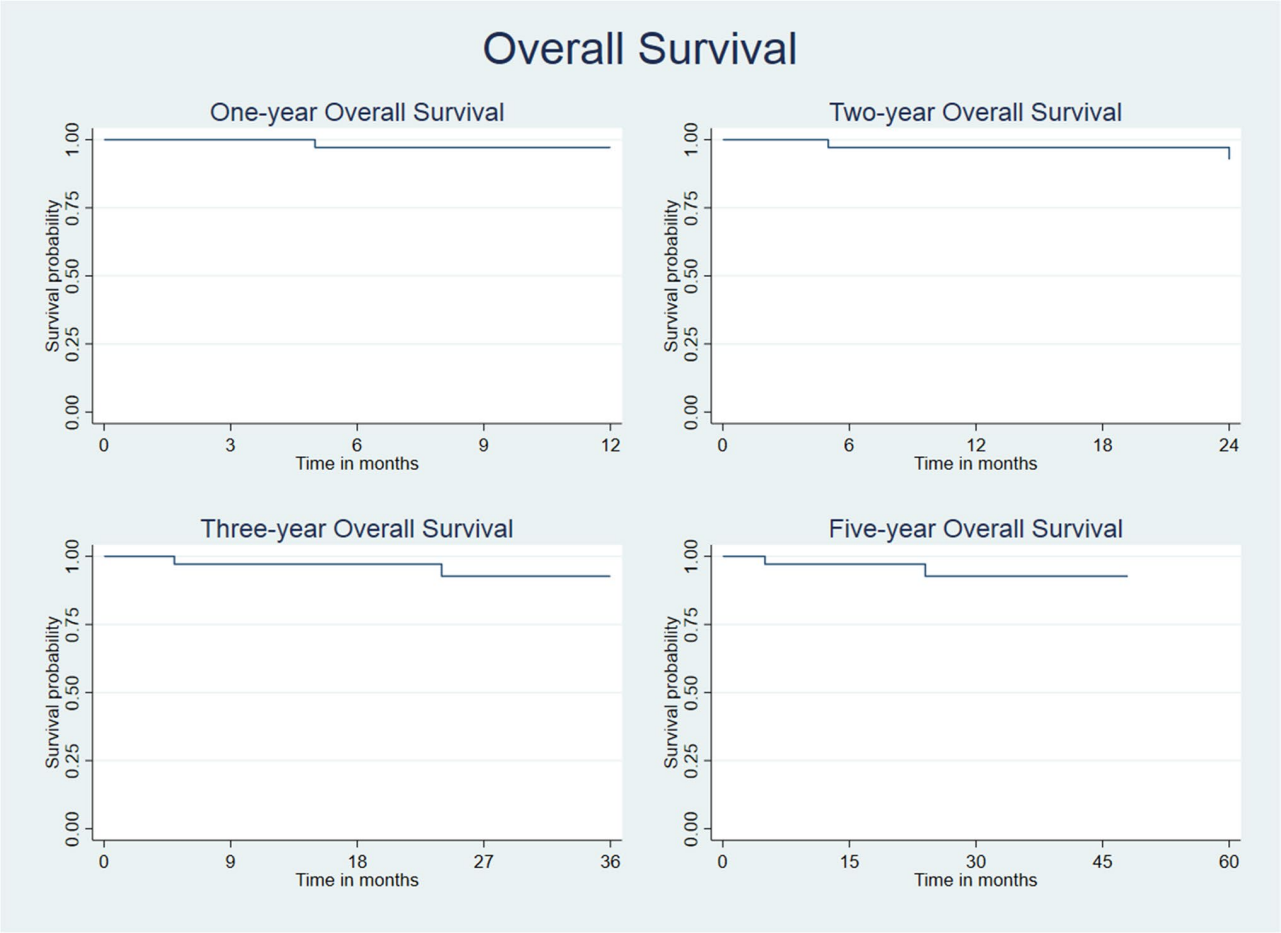
Kaplan–Meier analysis estimates overall survival rates over time

**Fig. 2:**
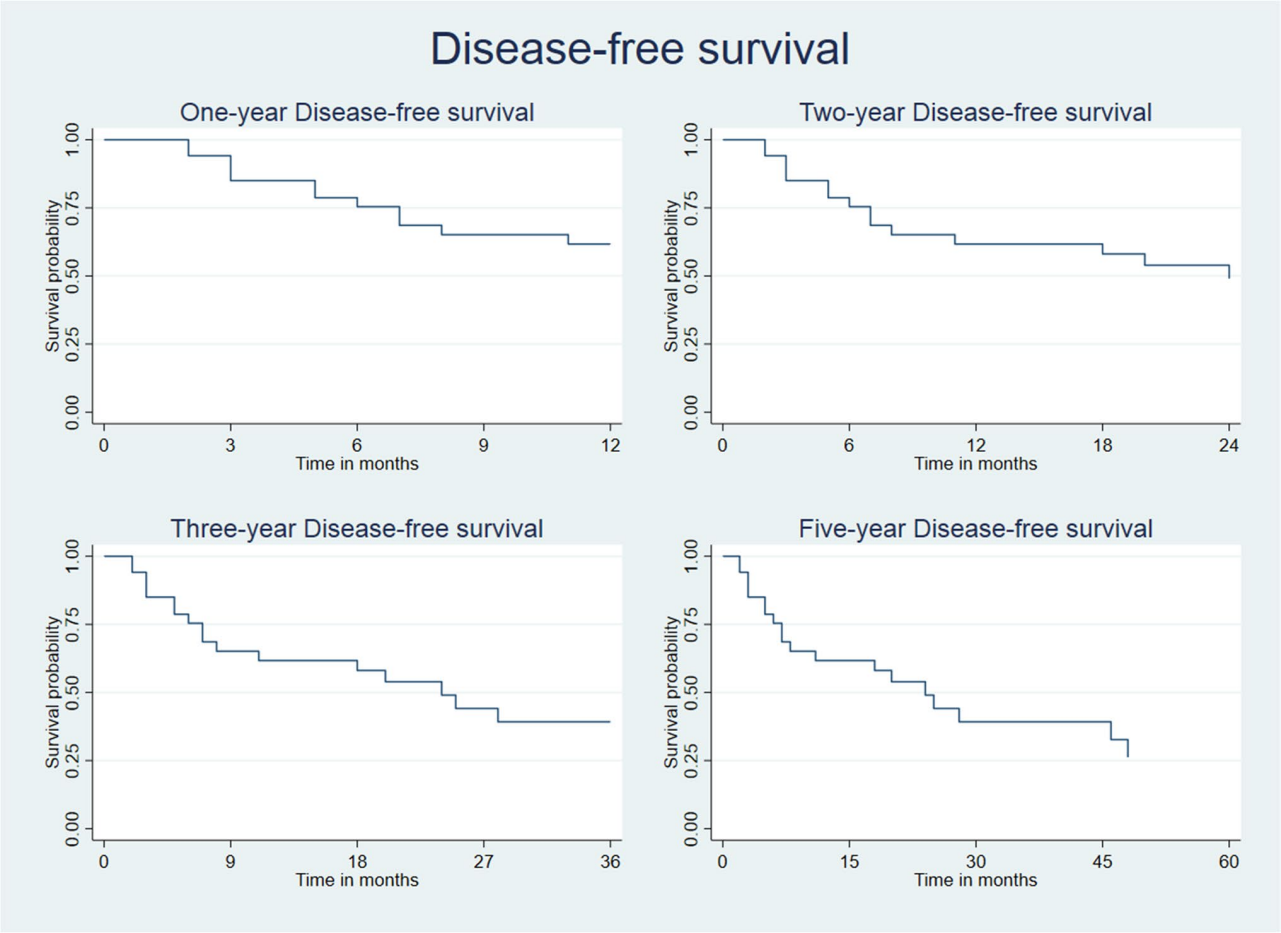
Kaplan–Meier analysis shows disease-free survival rates over time

The disease-free survival rates at 12, 24, 36, and 60 months were respectively 62.9%, 50.8%, 41.6%, and 29.7%. Overall survival rates and disease-free survival rates are represented in Fig. 1 and Fig. 2.

Five patients presented a second disease recurrence, but no statistically significant correlation with the abovementioned variables was found.

## Discussion

Based on the series of 5002 conjunctival tumors referred to an ocular oncology tertiary care center, malignant conjunctival tumors were found to be 30%. In that series, the ocular surface squamous neoplasia (OSSN), including conjunctival intraepithelial neoplasia (CIN) and squamous cell carcinoma (SCC), represented 14% of the total of conjunctival tumors, with SCC accounting for 9% of malignancies [1].

In the USA and Western Europe, most patients present with “classic OSSN” with typical limbal presentation and relatively benign clinical course. However, a minority of patients present more aggressively behaving tumors, with a higher rate of recurrence and associated ocular, periocular, and orbital invasion. In the current literature, only case reports or small case series of OSSC with periocular involvement and/or orbital invasion are reported.

In order to analyze a homogeneous sample, we selected 36 out of 618 patients with histologically proven conjunctival SCC with periocular involvement (T3 and T4 AJCC stage). Thus, 5.8% of cases represented advanced forms of OSCC. This result agrees with other reports, considering that intraocular invasion has been observed in 3% to 9% of cases of OSSN reported in the literature, whereas orbital invasion has been described in 1% to 6% [6–9]. Other authors reported that, among secondary orbital tumors originating from conjunctival malignancies, 81% of cases were identified as squamous cell carcinomas [10].

Tarsal involvement (T3 AJCC stage) and more generally higher AJCC stage, positive pathologic margins and higher pathologic grade were reported as strong predictors for more aggressive tumors [7, 11]. Although OSCC occurs predominantly in males [12, 13], we found an equal distribution in genders. None of the included cases showed HIV positivity, albeit invasive and aggressive variants of OSSC are often observed in HIV patients [8, 11, 13]. However, there were two cases of systemic immunosuppression, related to immunosuppressant therapy for organ transplantation and splenic disease respectively.

Analyzing 389 excised OSSN lesions, Galor et al. found that the 1-year recurrence rate was 10% and the 5-year recurrence rate was 21%, with a mean time to recurrence of 2.5 years. In our series, the overall recurrence rate was significantly higher (64% vs 21%) if compared with literature data, as would be expected for the kind of sample selected. Indeed, 31 patients presented a T3 stage disease at baseline, while 5 of them referred with a T4a stage and 1 presented a T4b stage.

Our results show that tumor anatomical characteristics at presentation, including multicentric pattern and inferior tarsus involvement, histological diagnosis of high-risk SCC, and positive surgical margins are significantly related to a higher risk of local recurrence. In particular, inferior tarsus involvement can be considered as a high-risk factor because the lower eyelid submucosal anatomical structures, tarsal plate, retractors, and fascial fibers of Lockwood’s ligament are probably more easily infiltrated, due to the lower development and thickness compared to similar submucosal superior eyelid structures and submucosal medial and lateral canthal tendons [14].

The type of treatment is also correlated with the recurrence rate [15–18]. Surgery alone (EB without adjuvant treatment) is significantly associated with a higher recurrence rate. Moreover, disease recurrence tends often to spread from the anatomical site of origin toward periocular tissues and orbital structures. In fact, five (22%) out of 23 recurred patients presented orbital invasion, with disease progression from the initial T3 stage. It cannot be ruled out that a further T3 stage stratification could better depict this spread and could also be a useful tool to define treatments and prognosis. According to other reports [11, 18], adjuvant topical therapy seems effective in decreasing recurrence rates, particularly in patients with positive margins, histological high-risk SCC, tarsal, and multicentric pattern anatomical involvement.

In conclusion, we suggest that in advanced OSCC, particularly when the inferior tarsus is involved and/or there is a multicentric disease, an aggressive and—where possible—radical surgery, consistent with the reconstruction needs, is strongly advisable. Customized adjuvant treatments should always be planned, including EBRT, brachytherapy, local, and also systemic chemotherapy. Cemiplimab, a PD1 inhibitor which was recently approved for the treatment of cutaneous SCC, has been suggested for OSCC patients and could be considered in the therapeutic strategy [19–21].

## Data Availability

Data and materials are available for consultation.
